# Peaking Industrial CO_2_ Emission in a Typical Heavy Industrial Region: From Multi-Industry and Multi-Energy Type Perspectives

**DOI:** 10.3390/ijerph19137829

**Published:** 2022-06-26

**Authors:** Haiyan Duan, Xize Dong, Pinlei Xie, Siyan Chen, Baoyang Qin, Zijia Dong, Wei Yang

**Affiliations:** 1 Key Lab of Groundwater Resources and Environment, Ministry of Education, Jilin University, Changchun 130021, China; yangwea@jlu.edu.cn; 2 College of New Energy and Environment, Jilin University, Changchun 130021, China; dongxz19@mails.jlu.edu.cn (X.D.); siyan21@mails.jlu.edu.cn (S.C.); qinby19@mails.jlu.edu.cn (B.Q.); zdong3@pride.hofstra.edu (Z.D.); 3 People’s Government of Daqiao Town, Jiangdu District, Yangzhou 225211, China; petrel1980@126.com

**Keywords:** industrial sector, CO_2_ emission, peak, influencing factor, LEAP model

## Abstract

Peaking industrial carbon dioxide (CO_2_) emissions is critical for China to achieve its CO_2_ peaking target by 2030 since industrial sector is a major contributor to CO_2_ emissions. Heavy industrial regions consume plenty of fossil fuels and emit a large amount of CO_2_ emissions, which also have huge CO_2_ emissions reduction potential. It is significant to accurately forecast CO_2_ emission peak of industrial sector in heavy industrial regions from multi-industry and multi-energy type perspectives. This study incorporates 41 industries and 16 types of energy into the Long-Range Energy Alternatives Planning System (LEAP) model to predict the CO_2_ emission peak of the industrial sector in Jilin Province, a typical heavy industrial region. Four scenarios including business-as-usual scenario (BAU), energy-saving scenario (ESS), energy-saving and low-carbon scenario (ELS) and low-carbon scenario (LCS) are set for simulating the future CO_2_ emission trends during 2018–2050. The method of variable control is utilized to explore the degree and the direction of influencing factors of CO_2_ emission in four scenarios. The results indicate that the peak value of CO_2_ emission in the four scenarios are 165.65 million tons (Mt), 156.80 Mt, 128.16 Mt, and 114.17 Mt in 2040, 2040, 2030 and 2020, respectively. Taking ELS as an example, the larger energy-intensive industries such as ferrous metal smelting will peak CO_2_ emission in 2025, and low energy industries such as automobile manufacturing will continue to develop rapidly. The influence degree of the four factors is as follows: industrial added value (1.27) > industrial structure (1.19) > energy intensity of each industry (1.12) > energy consumption types of each industry (1.02). Among the four factors, industrial value added is a positive factor for CO_2_ emission, and the rest are inhibitory ones. The study provides a reference for developing industrial CO_2_ emission reduction policies from multi-industry and multi-energy type perspectives in heavy industrial regions of developing countries.

## 1. Introduction

With the rapid development of society and economy, carbon dioxide (CO_2_) emissions from human activities have caused global climate change. In 2015, the Paris Agreement proposed the goal of keeping global temperature growth within 2 degrees Celsius, and the coordinated reduction of CO_2_ emissions is an effective way to achieve this goal [1]. A large part of CO_2_ emissions be produced from the rapid growth of industry in developing countries [2,3]. Since 2010, China has become the world’s largest CO_2_ emitter, accounting for 28.21% of the world’s total CO_2_ emissions [4,5]. China has pledged to peak CO_2_ emissions by 2030. From a regional perspective, CO_2_ emissions on the consumer side are mainly concentrated in the industrial sector, construction sector, transportation sector and urban household sector [6,7,8]. The industrial sector is the largest contributor to China’s CO_2_ emissions. The proportion of energy consumption in industrial sector accounts for more than 65% and the CO_2_ emissions of industrial sector is higher than a share of over 70% [9]. Peaking industrial CO_2_ emissions is critical for China to achieve its CO_2_ emission peaking target by 2030.

Industrial sector consists of multiple industries and involves multi-type energy consumptions. High CO_2_ emissions in the industrial sector stimulated scholars’ interest in studying the influencing factors for CO_2_ emissions from the industrial sector. In the energy-related industrial CO_2_ emissions study for Shanghai from 1994 to 2009, industrial growth and coal-type consumption had the most important effects on increasing industrial CO_2_ emission whereas energy efficiency played the most prominent role in reducing it [10]. Industrial activity and industrial scale were main driving force factor for industrial CO_2_ emission while energy intensity was the main factor decreasing emission in studies on CO_2_ emission in China’s industrial sector and China’s energy intensive industries [11,12]. Zhao et al. [13] investigated the carbon emission reduction effect of China’s industrial structure adjustment and revealed that upgrading of industrial structure can effectively realize carbon emission reduction. Feng et al. [14] and Lin et al. [15] respectively took Shanghai and Zhuhai as examples to explore the roles of economic growth, economic output value, urbanization, industrial structure, energy intensity and energy consumption types on CO_2_ emissions mitigation of industrial sector. Among the above influencing factors, most of studies confirmed that economic growth, economic scale, and population are the positive factors for CO_2_ emission, however, energy intensity, industrial structure and energy consumption types are the main contributors to mitigate CO_2_ emissions of industrial sector, which has been validated in many other studies [16,17,18,19,20].

It is essential to forecast the peak time and peak value of CO_2_ emission to effectively formulate relevant policies and achieve the long-term goal of CO_2_ emission mitigation for China [21]. According to previous studies, forecasting methods can be mainly divided into two types of models including top-down models, such as multi-objective optimization model [22], the regression on population, affluence, and technology (STIRPAT) model [23], Logarithmic Mean Divisia Index (LMDI) model [16], IPAT model [24], and bottom-up models such as the Long Term Energy Replacement Program (LEAP) model [25,26] and input-output model [27]. For example, Yu et al. [21] used a new economic-carbon emission-employment multi-objective optimization model to analyze China’s CO_2_ emission trajectory and indicated that CO_2_ emission of energy consumption would peak between 2022 and 2025. Duan [28] applied STIRPAT model to predict the peak of the industrial CO_2_ emission and analyzed the driving factors in four scenarios in Jilin Province during 2015–2050. The above two studies both used top-down models, which made them lack of discussion on the contribution of different industries and energy type to peaking CO_2_ emission. Zhang et al. [25] used STIRPAT and LEAP models to analyze the data from five sectors of Yunnan Province, including power, transportation, construction, steel and chemical industries and indicated that Yunnan’s CO_2_ emission would peak at 2 million tons during 2024–2028. The calculation results of the LEAP model are considered to be more accurate given more detailed parameters and scenario setting. Mi et al. [27] applied an integrated model of economy and climate is developed based on input-output analysis to assess China’s peak of CO_2_ emission involving 33 industries under five scenarios, demonstrating that China’s CO_2_ emissions would peak as early as 2026. The above studies used bottom-up model presented more industry-specific CO_2_ emission predictions. In addition, we list some previous studies that used different methods and set multiple scenarios to predict the relevant CO_2_ emissions of the industrial sector, as shown in Table 1. Through the comparison of these studies, it is found that although these studies fully simulated CO_2_ emissions from holistic industrial sector [28,29,30]. major manufacturing industries [31], industrial sub-sectors [32,33], the steel industry [34] and the China’s total CO_2_ emissions by 33 industries [27], there is a lack of specific-industry CO_2_ emission forecasts that break down the industrial sector into 41 industrial industries considering multi-energy types.

The LEAP model is a popular bottom-up model for assessing future energy consumption and CO_2_ emissions. It has the characteristics of powerful accounting ability, flexible modeling parameter setting, and analysis of rich technical specifications and end-use details [35]. For example, Liu et al., employed the LEAP model to estimate the energy consumption, CO_2_ and air pollutant emissions of China’s transport sector between 2010 and 2050 under four scenarios: Business as Usual (BAU), Energy Efficiency Improvement (EEI), Transport Mode Optimization (TMO), and Comprehensive Policy (CP) [36]. LEAP model was also utilized to simulate six energy sectors-related GHG emissions under three scenarios in Ningbo city, and forecast greenhouse gas emissions in China’s tourist industry under two scenarios [37,38]. Based on the advantages of flexible parameters and scenario settings, the LEAP model is very suitable to be used for predicting emissions in various sectors and industries.

Due to the vast territory of China, there are obvious differences in the industrial structure and resource endowment of various regions [39]. Because the excellent resource endowment in some regions provides the foundation for the development of heavy industry, these regions usually focus on the development of heavy industry dominated by energy-intensive industries, forming an energy-dependent industrial structure [40]. The economic development of these heavy industrial regions mainly relies on fossil fuels, which emits a large amount of CO_2_ emissions [41]. Heavy industrial regions also have huge CO_2_ emissions reduction potential, and green transitions in heavy industrial regions play a vital role in peaking industrial CO_2_ emissions and achieving sustainable development [42]. However, from the perspective of economic development in these regions, restricting the development of heavy industries dominated by energy-intensive industries may greatly hinder regional economic development. Then, while maintaining the rapid growth of the regional economy, getting rid of the dependence on the original energy-intensive industrial structure and reducing regional energy consumption and CO_2_ emissions bring new challenges to these regions.

Previous studies have confirmed that the industrial sector is extremely important in peaking regional CO_2_ emissions. Meanwhile, reducing CO_2_ emissions in heavy industrial regions plays a vital role in peaking CO_2_ emissions in China’s industrial sector. Currently, some existing studies have predicted peak CO_2_ emissions of the industrial sector from the perspective of multiple industrial subsectors, but the industrial sector was usually divided into less than ten subsectors. A sufficient discussion of peak CO_2_ emissions of all industries of the industrial sector is lacking. In addition, forecasting the peak CO_2_ emissions of the industrial sector in the heavy industrial region is also rough, and there is a lack of exhaustive discussions by industries to reflect the detailed CO_2_ emissions and energy consumption structure of each industry.

This study selects Jilin Province, a typical heavy industrial regions, as the research object. This region has a high proportion of heavy industry energy consumption and the industrial value added is 32.4% of the GDP in Jilin [43,44,45,46,47,48,49,50,51]. Jilin confronts the dual dilemma of economic development and CO_2_ emission reduction. According to Industrial Classification for National Economic Activities of China, the industrial sector consists of 41 industries covering a wide range of energy consumption types [52]. We utilize LEAP model to predict peak CO_2_ emission and the future trends of CO_2_ emission in industrial sector from 2018 to 2050 in four scenarios. LEAP model integrates 41 industrial industries and 16 types of energy and includes four scenarios, namely business-as-usual scenario (BAU), energy-saving scenario (ESS), energy-saving and low-carbon scenario (ELS) and low-carbon scenario (LCS). Then, this model is applied to analyze the driving degree of four influencing factors on the peak value and time of CO_2_ emission in industrial sectors. The result of the peak CO_2_ emissions of industrial sector and the peak CO_2_ emissions of each industry provide a reference for Jilin to develop CO_2_ emissions reduction strategies and indicate directions for follow-up efforts. In addition, this study also aims to provide a reference for industrial CO_2_ emission reduction from multi-industry and multi-energy type perspectives in heavy industrial regions of developing countries around the world.

## 2. Materials and Methods

**Figure 1:**
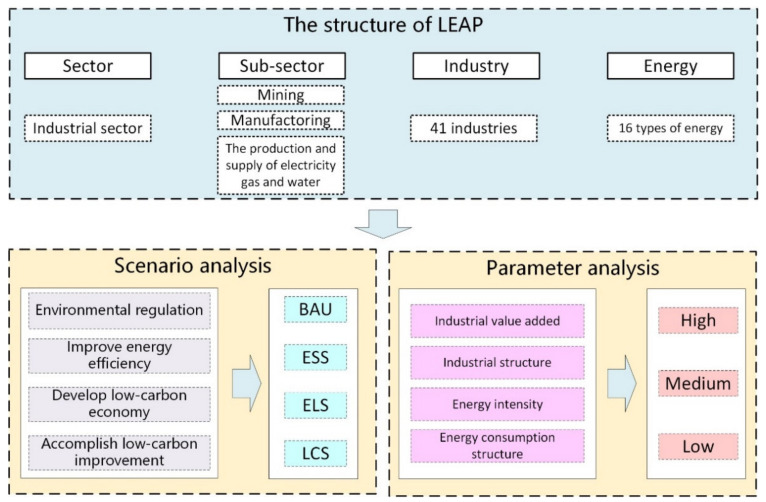
The research framework.

The research framework of this study is divided into three parts as shown in Figure 1. Firstly, since industrial sector involves multiple types of energy consumption and more than 40 industries, this study incorporates 41 industries and 16 types of energy into the LEAP model in industrial sector. Secondly, four scenarios, namely a business-as-usual scenario (BAU), an energy-saving scenario (ESS), an energy-saving and low-carbon scenario (ELS) and a low-carbon scenario (LCS) are set for simulating the trend of CO_2_ emission during 2018–2050. Finally, the method of variable control is utilized to explore the degree and the direction of influencing factors of CO_2_ emission in 48 sub scenarios.

### 2.1. LEAP Model

Since the nature and characteristics of production processes in different industries of the industrial sector vary, CO_2_ emissions of different industries are also diverse, which results differences in CO_2_ emission reduction targets of industries [53]. Based on the advantages of flexible parameters and scenario settings, the LEAP model is very suitable to be used for predicting the peak CO_2_ emissions of the industrial sector from multi-industry and multi-energy type perspectives.

Based on the LEAP framework, this study established a four-level activity with reference to Industrial Classification for National Economic Activities of China, in which the CO_2_ emission factor is associated with activity level 4. The content of each activity level is as follows: (1) activity level 1 (sector): industrial sector; (2) activity level 2 (3 sub sector): mining, manufacturing, and production and supply of electricity, gas and water; (3) activity level 3 (41 industry): oil and gas exploration, pharmaceutical manufacturing, the production and supply of electricity and heat, etc. (4) activity level 4 (16 types of energy): raw coal, coking coal, gasoline, diesel oil, heat, electricity, etc.

**Figure 2:**
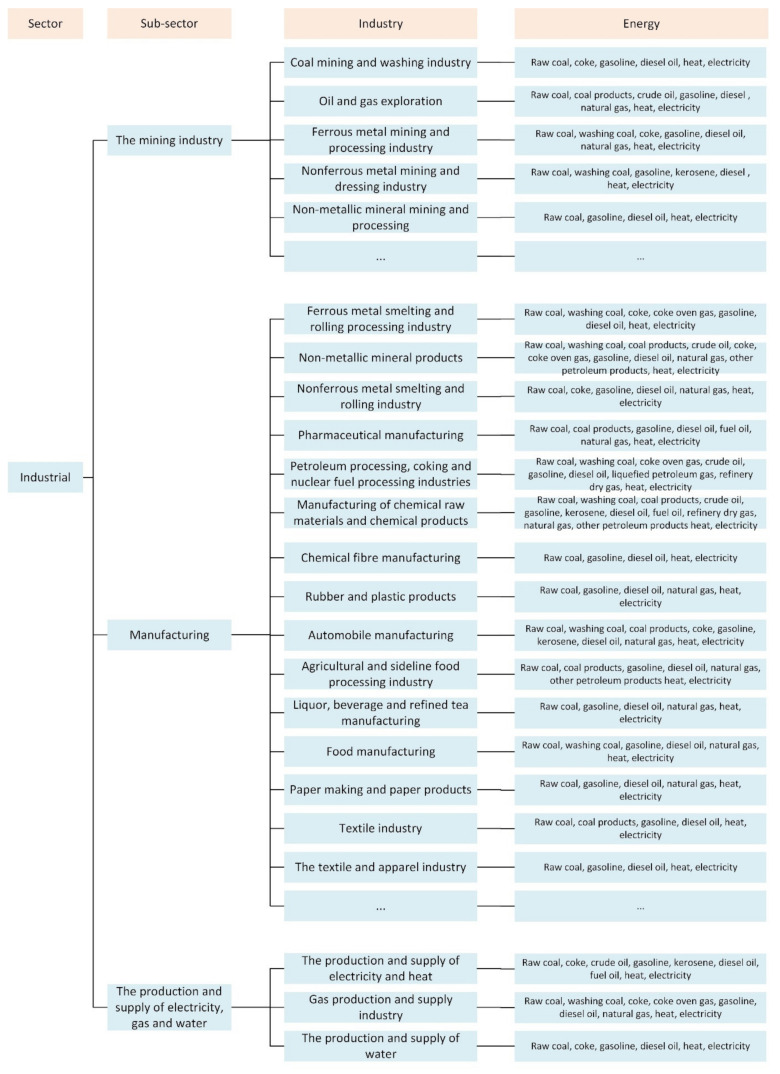
Constructed dendritic structure of the industrial sector in the LEAP model.

In level 3, the mining includes 7 industries such as mining and washing of coal industry, extraction of petroleum and natural gas industry, mining and processing of ferrous metals ores industry, etc. Manufacturing includes 30 industries such as agricultural and sideline food-processing industry, food manufacturing industry, automobile manufacturing industry, etc. The production and supply of electricity, gas and water comprise the production and supply of electricity and heat, the production and supply of gas and the production and supply of water. The existing industrial types are fully considered in the model construction. The specific model framework is shown in Figure 2.

### 2.2. Calculation Method of CO_2_ Emission

In LEAP model, CO_2_ emission can be calculated according to the following process.

(1) $$C_{ij}=E_{ij}\times F_{j}$$

where, *C_ij_* represents the CO_2_ emission from different industrial industries and energy types (Million tons); *i* is industrial industries (*i* = 1, 2, …, *n*); *j* represents energy types (*j* = 1, 2, …, m); *E* is the energy consumption (tons of coal-equivalent); *F* represents the energy coefficient of CO_2_ emission.

(2) $$E_{ij}=\overset{}{I}\times P_{i}\times T_{i}\times U_{ij}$$

where, *E* is the energy consumption (tons of coal-equivalent); *i* is industrial industries (i = 1, 2, …, *n*); *j* represents energy types (*j* = 1, 2, …, m); *I* represents the industrial value added (10^3^ yuan); *P* represents the proportion of the value added of each industry in the industrial value added (%), which shows the industrial structure; *T* is the energy intensity of each industry (tons of coal-equivalent/10^3^ Yuan (tce/10^3^ Yuan)), which indicates the technological progress of each industry and the level of energy efficiency; *U* is the proportion of all kinds of energy consumption in each industry(%), which shows the energy consumption types.

(3) $$F_{j}={NCV}_{j}\times {CC}_{j}\times O_{j}\times \frac{44}{12}$$

where, *F* is the direct CO_2_ emission coefficient of fossil energy consumption; *j* is for different fossil energy types; *NCV_j_* is the average low calorific value of energy *j* (Kilojoules/Kilogram (kJ/kg)); *CC_j_* is carbon content per unit calorific value of energy *j* (Tons of carbon/Terajoule (tC/TJ)); *O_j_* is the carbon oxidation rate of energy *j* (%); 44/12 is the conversion coefficient of CO_2_ emission. The 14 types of fossil energy consumption are indicated in Table 2. The other two types of energy consumption are electricity consumption and heat consumption. This studies includes 16 types of energy consumption.

CO_2_ emission factor of electricity consumption

(4) $${EE}_{t}=\frac{{TE}_{t}}{{EP}_{t}}\times \frac{{EC}_{t}}{{EC}_{et}}$$

where, *EE_t_* is the CO_2_ emission factor of electric power consumption in the *t* year; *TE_t_* is the CO_2_ emission from thermal power generation in the *t* year; *EP_t_* is total electricity generation in the *t* year; *EC_t_* is the total electricity consumption in the *t* year; *EC_et_* is the total power terminal energy consumption in the *t* year.

CO_2_ emission factor for heat consumption

(5) $${HE}_{t}=\frac{{HP}_{t}}{{HC}_{et}}$$

where, *HE_t_* is the CO_2_ emission coefficient of heat consumption in the *t* year; *HP_t_* is the CO_2_ emission from heat production in the *t* year; *HC_et_* is the total energy consumption of heat terminal in the *t* year.

(6) $$\overset{}{C}=\sum C_{ij}=\;\overset{}{I}\times P_{i}\times T_{i}\times U_{ij}\times {NCV}_{j}\times {CC}_{j}\times O_{j}\times \frac{44}{12}$$

where, *C* represents the total CO_2_ emission (Million tons); *C_ij_* represents the CO_2_ emission from different industrial industries and energy types (Million tons); *i* is industrial industries (i = 1, 2, …, *n*); *j* represents energy types (*j* = 1, 2, …, m).

## 3. Case Study

### 3.1. Study Area

Jilin is a heavy industrial region located in Northeast China. Heavy industry accounts for 82% of the total industrial energy consumption in Jilin [51]. Due to its excellent resource endowment and unique historical development opportunities, Jilin had developed into one of the largest industrial regions during the First Five-Year Plan. As a production region for automobiles, chemicals, crude oil, and steel, Jilin made a distinguished contribution to the economic development of China before the 1970s. At present, Jilin has a complete range of industries, and the pillar industries of Jilin are still automobile, petrochemical and agricultural product processing industry. Among them, the value added of transportation facilities manufacturing industry has always accounted for the highest proportion of industrial value added. The value added of low-emission, high-value-added industries such as automobile manufacturing and electronic equipment manufacturing such as computers and communications maintained steady growth. Chemical raw materials and chemical product manufacturing, ferrous metal smelting and rolling processing industry, non-metallic mineral product industry and electricity, heat production and supply industry are the most energy-consuming industries in Jilin. Meanwhile, energy consumption types in Jilin are dominated by coal, oil, heat, electricity and natural gas. With the continuous development of global economy, Jilin’s economic development gradually falls behind the average level of economy in China. Unreasonable industrial structure, outdated technology and equipment, and high emission amount have been becoming more prominent. In this context, the target of peak CO_2_ emission has brought the double pressure of economic development and low-carbon sustainable development for Jilin. This paper chooses Jilin as the research area to provide reference for other heavy industrial regions in developing countries confronting the same dilemma.

### 3.2. Scenarios

This paper sets up four scenarios, including business-as-usual scenario (BAU), energy-saving scenario (ESS), energy-saving and low-carbon scenario (ELS) and low-carbon scenario (LCS), to analyze mitigating CO_2_ emissions of the industrial sector. Scenario setting is mainly considered according to technological upgrading and innovation, industrial structure change, and energy consumption structure improvement. The scenarios for this study are set from 2018 to 2050, as the Paris Agreement requires participating countries to report long-term low-carbon development plans before 2050. Four scenarios are described in Appendix A. Transportation equipment manufacturing industry and agricultural and sideline food processing industry are taken as examples.

#### 3.2.1. Business-as-Usual Scenario (BAU)

Business as usual scenario (BAU) is designed to simulate CO_2_ emissions of the industrial sector given current policies and technological level in 2018, and the parameters in this scenario keep stable during 2018 to 2050. The 13th Five-Year Development Plan for Industry of Jilin Province [57] mentioned that the industrial value added of Jilin Province would increase at an average annual rate of not less than 6%. The industrial added value of the automobile industry, the petrochemical industry and the agricultural product processing industry has an average annual increase of 7.6%, 7.6% and 6%, separately. The specific performance of the BAU scenario is rapid industrial development and rapid industrial added value growth. Traditional industrial industries with high energy dependence, high energy intensity and limited energy efficiency account for a large proportion of industrial added value. Therefore, the BAU scenario is the maximum boundary that can be achieved when economic development is fully promoted without considering ecological and environmental benefits.

#### 3.2.2. Energy-Saving Scenario (ESS)

Unlike the BAU scenario that focuses on industrial economic development, the ESS scenario reflects the economic development, energy consumption and CO_2_ emission status after improving energy utilization efficiency and popularizing energy saving technologies, based on industrial value added, industrial structure, energy intensity and energy consumption types. Specifically, energy intensity of the industrial sector is reduced; energy efficiency is improved; the proportion of clean energy in terminal energy consumption is increased; the proportion of traditional fossil energy is decreased. Meanwhile, the growth rate of industrial value added and the state of industrial structure in the ESS scenario and the BAU scenario are the same.

#### 3.2.3. Energy-Saving and Low-Carbon Scenario (ELS)

In Energy-saving and low-carbon scenario (ELS), low-carbon economy is vigorously promoted because of energy conservation and emission reduction policies. In the initial stage setting of the ELS, industrial value added, industrial structure, energy intensity and energy consumption types should refer to the state of them in the ESS. Then, the ELS gradually strengthens the development of low-carbon economy under the support of energy conservation and emission reduction policies, for example, promoting the use of clean energy, changing the mode of economic growth, and vigorously developing low energy consumption and high value-added industries.

#### 3.2.4. Low-Carbon Scenario (LCS)

The low-carbon scenario (LCS) is to comprehensively implement the low-carbon economy, fully accomplish the sustainable development of economy and environment, accelerate the technological progress and the adjustment of industrial structure, and further adjust the economic growth mode. Specifically, the growth rate of industrial value added slows down further and the proportion of the industrial sector declines further. The energy intensity of all industries continues to decline and the level of energy efficiency is further improved. The industrial structure is further optimized, and the proportion of low-pollution and high value-added industries is constantly increasing. The energy consumption types are further adjusted, and the proportion of clean energy basically stabilizes at a relatively high level. LCS demonstrates the maximum reduction that is available to balance the economy and emission.

### 3.3. Parameter Setting

In order to explore peak CO_2_ emission in Jilin’s industry, the influencing factors are set to high mode, medium mode, and low mode, and appropriate coefficients need to be set. Corresponding to the four levels of LEAP model, four influencing factors are selected in this study, which are industrial value added, proportion of the value added of each industry in the industrial value added, energy intensity of various industries and energy consumption types of various industries. According to the changing modes of each factor (i.e., high mode, medium mode, or low mode), the four scenarios including BAU, ESS, ELS and LCS are composed by these four influencing factors, as shown in Table 3. Based on the 14th Five-Year Plan of Jilin, the parameters are shown in Appendix A, Appendix B, Appendix C, Appendix D, Appendix E.

### 3.4. Data

The data of 41 industries and 16 types of energy consumption are sourced from the Jilin Statistical Yearbook [43,44,45,46,47,48,49,50,51], covering industrial value added, and industrial sector terminal energy consumption from 2011 to 2018. As for industrial energy consumption CO_2_ emission, this research calculates CO_2_ emissions adopting the CO_2_ emission calculation method and CO_2_ parameters of different energy types that recommended by the Intergovernmental Panel on Climate Change (IPCC) [58]. Correlation coefficient of fossil energy are sourced from Guidelines for Compiling Provincial Greenhouse Gas Inventories (NDRC [2011] No. 1041) [46], China Energy Statistical Yearbook 2020 [47] and General Principles for Calculation of Comprehensive Energy Consumption (GB/T 2589-2020) [48].

## 4. Results

### 4.1. Analysis of CO_2_ Emission Reduction Potential of Industrial Sector

**Figure 3:**
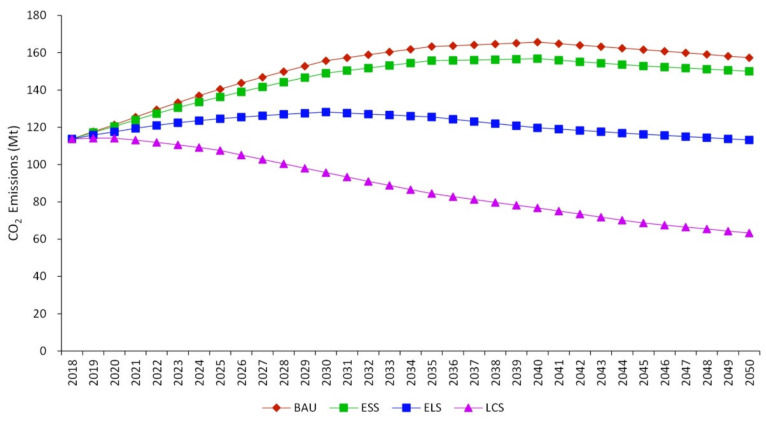
CO_2_ emission from industrial sectors in Jilin Province in different scenarios.

In the four scenarios, the forecast results of CO_2_ emission from industrial sectors in Jilin from 2018 to 2050 are shown in Figure 3 in the BAU, ESS, ELS, and LCS scenarios, the CO_2_ emission peaks are 165.65 Mt, 156.80 Mt, 128.16 Mt, and 114.17 Mt, respectively, and the corresponding peak times are 2020, 2030, 2040, and 2040, respectively. The earliest peak that are 10 years earlier than the peak in the ELS, and 20 years earlier than the peak in the ESS and BAU will occur in the LCS in 2020. Compared with previous study [25], the Jilin’s peak of industrial CO_2_ emission in BAU is obviously earlier than that calculated by STIRPAT method in Jilin. In peak years, the peaks in ESS, ELS, and LCS decreased by 5.34%, 22.63%, and 31.08%, respectively, compared to the peak in BAU. In 2050, the corresponding CO_2_ emission of BAU, ESS, ELS and LCS are 157.32 Mt, 150.09 Mt, 113.21 Mt and 63.29 Mt, respectively, which are 94.97%, 95.71%, 88.33% and 55.43% of the CO_2_ emission at the peak year. The CO_2_ emission of both LCS and ELS will be decreased significantly in 2050, which indicates that LCS and ELS have excellent long-term sustainable CO_2_ emission reduction potential, and the decline rate in LCS is significantly better than that of the other three scenarios. From the perspective of accumulation, the cumulative CO_2_ emission from 2018 to 2050 are 5003.58 Mt, 4798.97 Mt, 3995.64 Mt and 2925.67 Mt respectively, corresponding to BAU, ESS, ELS and LCS. The CO_2_ emission in BAU is 1.7 times of that in LCS and 1.3 times of that in ELS. The result shows that LCS has the best CO_2_ emission reduction potential both in the long term and in the cumulative perspective.

### 4.2. Optimal Scenario Selection

Through the prediction of future CO_2_ emission, the result shows that the LCS is significantly better than the other three scenarios in terms of peak time and peak value. From a long-term and cumulative point of view, the LCS scenario still has significant advantages. In 2050, the minimum CO_2_ emission occurring in the LCS would be only 0.56 of that in the nearest ELS. From a cumulative point of view, from 2018 to 2050, the cumulative reduction of CO_2_ emission in LCS is 1069.98 Mt compared with that in ELS. Industrial structure, industrial energy intensity, industrial energy consumption types and other aspects of the development have reached a relatively complete level in LCS. However, the LCS requires such high technical level that is difficult to achieve in a short period and necessitates the high socioeconomic cost that is a heavy burden for the future development of Jilin. ELS can be used as a transition stage, and its predicted results are basically in line with the general law of industrial development. Meanwhile, CO_2_ emission in ELS will reach the peak in 2030. Combined with the actual development situation, ELS scenario should be taken as the optimal scenario at the present stage in Jilin, in order to ease the conflict between CO_2_ emission reduction and economic development. After the technical level is gradually improved, LCS scenario may replace ELS as the optimal one in the future.

### 4.3. Multi-Industry CO_2_ Emission

#### 4.3.1. Multi- Industry CO_2_ Emission under the BAU Scenario

**Figure 4:**
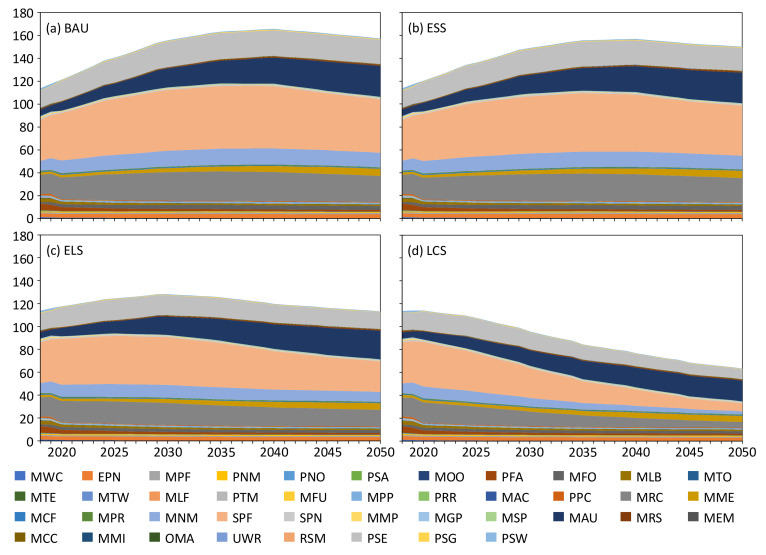
CO_2_ emissions of industries in four scenarios (million tons). The full name of the industry code is in Appendix F.

Under the BAU scenario, the CO_2_ emission peak of the industrial sector in Jilin is 2040. CO_2_ emissions of high emission industries such as SPF (ferrous metal smelting and rolling processing industry), MRC (chemical raw material and chemical products manufacturing industry), MNM (non-metallic mineral products industry), and PSE (electricity, heat production and supply industry) will continue to increase after 2018 and peak in 2035, as illustrated in Figure 4. CO_2_ emissions of the industries with low-emission and high-value-added such as MAU (automotive manufacturing) and MCC (computer, communications and other electronic equipment manufacturing) will continue to grow between 2018 and 2050. Among them, MAU (automobile manufacturing), as an existing advantageous industry and a key industry for future development in Jilin, will increase its CO_2_ emissions by 484% by 2050, far more than other industries. For MTE (textile industry), MLB (wine, beverage and refined tea manufacturing industry) and other industries, their total CO2 emissions are small (mostly less than 0.5 megaton), and the energy consumption types are mainly electricity. With the increase of the proportion of clean electricity, the emissions of these industries show a slow decline trend.

#### 4.3.2. Multi-Industry CO_2_ Emission under the ESS Scenario

Under the ESS scenario, the CO_2_ emission peak of the industrial sector in Jilin is 2040. Although the industrial energy efficiency in ESS scenario is improved on the basis of BAU scenario, the change trend of CO_2_ emissions in the two scenarios is basically the same because the energy consumption types and industrial structure have not been adjusted significantly, as described in Figure 4. The CO_2_ emissions of all industries in ESS scenario decreased compared with the data of corresponding years in BAU scenario.

#### 4.3.3. Multi-Industry CO_2_ Emission under the ELS Scenario

Under the ELS scenario, the CO_2_ emission peak of the industrial sector in Jilin is 2030. Compared with BAU and ESS, the industrial sector of Jilin has made significant adjustments in industrial structure under ELS. First, by restrict the development of high-emitting industries. The CO_2_ emissions of SPF (ferrous metal smelting and rolling processing industry), MRC (chemical raw materials and chemical products manufacturing), MNM (non-metallic mineral products industry), PSE (electric power and heat production and supply industry) will experience a small degree of increase (about 5%) during the 14th Five-Year Plan period, and peak in 2025, followed by a year-on-year decrease in emissions, as shown in Figure 4. Second, the development of low-emission, high-value-added industries will be supported, and the CO_2_ emissions of industries such as MAU (automobile manufacturing) and MCC (computer, communications and other electronic equipment manufacturing) will continue to grow.

#### 4.3.4. Multi-Industry CO_2_ Emission under the LCS Scenario

Under the LCS scenario, the CO_2_ emission peak of the industrial sector in Jilin is 2020. Under the low-carbon scenario, the industrial sector will adopt strict industrial structure adjustment and energy consumption types adjustment measures to reduce emissions. At the industry level, compared with ELS scenario, the CO_2_ emissions of high-emitting industries in LCS scenario will be strictly restricted after 2020, showing a decreasing trend gradually. In particular, SPF (ferrous metal smelting and rolling processing industry), MRC (chemical raw material and chemical products manufacturing) and PSE (electric power and heat production and supply industry) are the major contributors to Jilin’s industrial emission reduction, with cumulative reductions of 33.6%, 21.5% and 15.3% respectively by 2050. For industries such as MAU (automobile manufacturing), MCC (computer, communication and other electronic equipment manufacturing) and other industries, the energy consumption types are dominated by electricity, as indicated in Figure 4. The rapid cleaning up of the energy mix will reduce the emissions growth caused by the economic development of the industries, so the CO_2_ emissions of these industries have declined compared to ELS and will continue to plateau after 2040.

### 4.4. CO_2_ Emission of Industrial Sector from Multi-Energy Types in Four Scenarios

**Figure 5:**
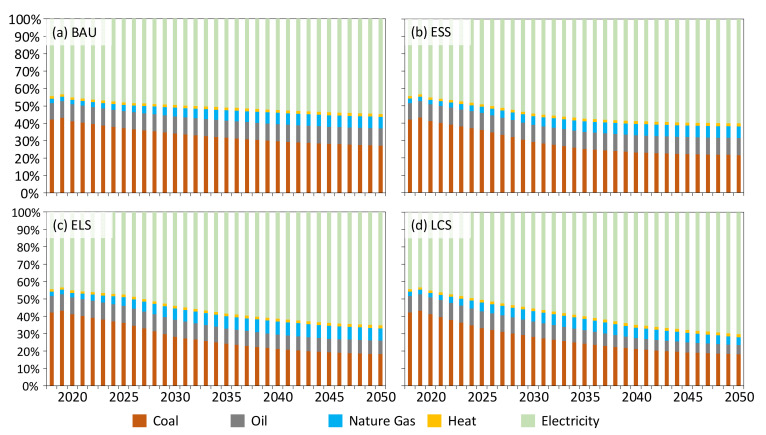
CO_2_ emissions from multi-energy types in four scenarios.

Under the BAU scenario, the proportion of CO_2_ emissions from coal consumption in industrial sectors in Jilin is decreasing year by year, reaching 29.64% in 2040. The CO_2_ emissions of the share of electricity and natural gas will increase significantly, reaching 52.35% and 6.54% respectively by 2040. The proportion of CO_2_ emission from oil consumption and heat consumption rises slowly, reaching 9.85% and 1.62%, respectively, in 2040, as shown in Figure 2. Under the ESS scenario, the energy consumption types are initially adjusted. Compared with BAU, the CO_2_ emissions of coal consumption under ESS decreased significantly during 2020–2030, and gradually became regionally stable after 2030. In contrast, the CO_2_ emissions of electricity consumption will increase rapidly between 2020 and 2030, and will remain stable in the same region after 2030. In ELS, as industrial emissions peak earlier, a more rapid transition of the energy consumption types is required to achieve the peaking target. Therefore, before the peak year (2030), the proportion of industrial coal consumption emissions will decline faster, and the proportion of CO_2_ emissions from electricity consumption is also growing faster. In addition, the CO_2_ emissions of the share of oil consumption and natural gas consumption will also slowly decline after 2030. Under LCS, the peak time is further advanced to 2020, and the magnitude and speed of energy consumption structure adjustment are further accelerated on the basis of ELS. The CO_2_ emissions of the share of fossil energy consumption such as coal, oil and natural gas have declined rapidly, and the degree of industrial electrification has increased gradually. By 2050, the CO_2_ emissions of the share of electricity consumption will reach 70.3%, while the CO_2_ emissions of the share of fossil energy consumption will be 28.0%. CO_2_ emissions from multi-energy types in four scenarios as pictured in Figure 5.

### 4.5. Analyze Influencing Factor of CO_2_ Emission from Industrial Sector

Using the method of control variables, this paper changes the change rate of one influencing factor into low rate, medium rate and high rate, in four scenarios, successively. In the premise, the change rate of other influencing factors remains unchanged, so as to obtain 48 sub scenarios. In the 48 sub scenarios, the driving degree of each influencing factor on the peak are quantitatively analyzed with the time and the value of the peak as the reference, as pictured in Table 4.

#### 4.5.1. Comparative Analysis of Peak Value

**Figure 6:**
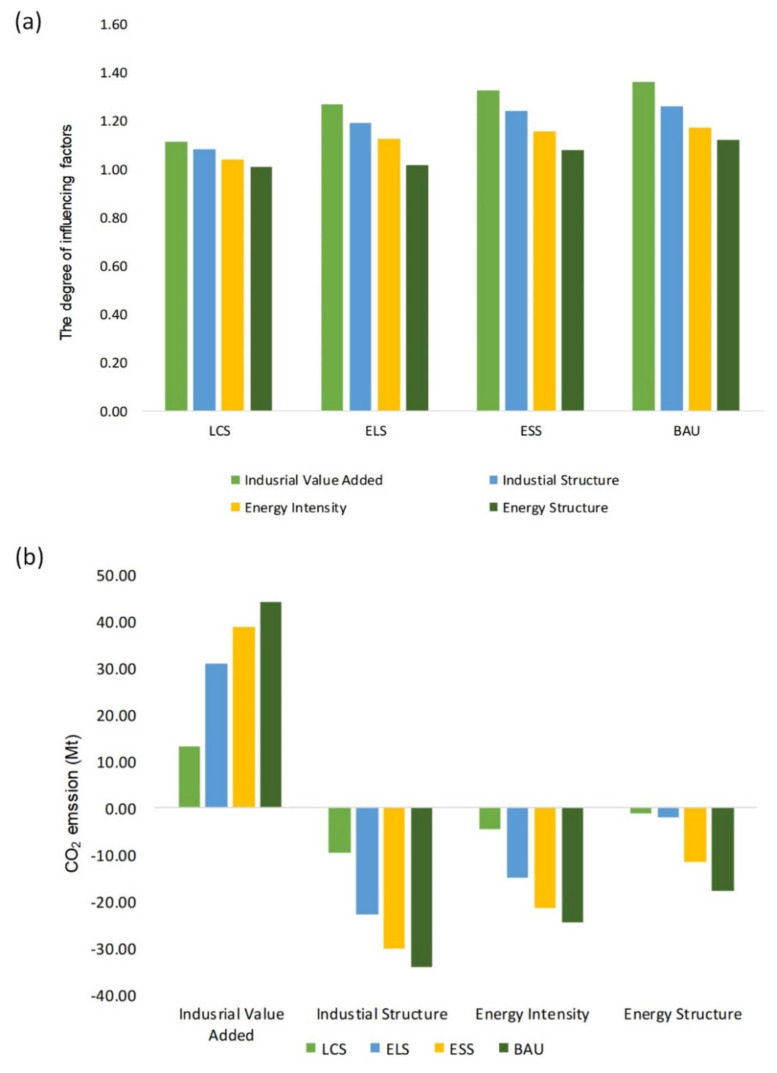
(**a**) The driving degree of four factors in four scenarios. The driving degree is determined by the ratio of maximum peak value to minimum peak value in high, middle and low modes. (**b**) The change value of four factors from high rate to low rate in four scenarios.

Four influencing factors, including industrial added value, the proportion of industrial added value in industrial added value (industrial structure), energy intensity and energy consumption types of each industry, have different degrees of influence on CO_2_ emission peak. In the four scenarios, the influence degree of these factors all shows the trend: industrial added value > industrial structure > energy intensity > energy consumption types of each industry. Figure 6 shows the driving degree of impact factors from low change rate to high change rate on CO_2_ emission in the four scenarios. The driving degree of four factors is determined by the ratio of maximum peak value to minimum peak value in high, middle and low modes. The influence of the four factors on the peak is consistent in the four scenarios, as shown in Figure 6. Taking ELS as an example, the influence degree of the four factors is as follows: industrial added value (1.27) > industrial structure (1.19) > energy intensity of each industry (1.12) > energy consumption types of each industry (1.02). The corresponding changes of the four influencing factors from low rate to high rate are industrial added value (30.70 Mt), industrial structure (−22.99 Mt), energy intensity of each industry (−15.01 Mt) and energy consumption types of each industry (−2.08 Mt), as shown in Figure 6.

#### 4.5.2. Comparative Analysis of Peak Time

From the perspective of the peak, the study analyzes driving degree of each influencing factor to the peak time of industrial energy consumption CO_2_ emission. The four influencing factors have various-degree effects on the peak time in the four scenarios. Taking ELS as an example, only two factors from low mode to high mode, including industrial value added and energy intensity of various industries, can affect the peak time in ELS. Industrial value added is also the most significant factor to change the peak time that will delay 19 years to 2040 when industrial value added varies from low mode to high mode. The higher the rate of industrial value added, the later the peak time appears. The peak time will be brought forward by 2 years when energy intensity of various industries changes from low mode to high mode. Therefore, maintaining steady economic growth and improving the application and promotion of low-carbon technologies are effective means to advance the peak time.

## 5. Discussion and Policy Implications

The industrial value added is the most significant influencing factor for the increase of CO_2_ emission. In recent years, the industrial value added of Jilin has increased steadily. Due to the characteristics of high industrial emission and high energy consumption in Jilin, the industrial development will inevitably bring the pressure of CO_2_ emission. With the improvement of low-carbon city, its sensitivity to the change of industrial value added becomes less and less. In addition, the other three influencing factors all have a negative driving effect on the peak of CO_2_ emission, among which the industrial structure has the most significant negative driving effect.

The above results reveal that the larger energy-intensive industries, such as chemical raw materials and chemical products manufacturing, ferrous metal smelting and rolling processing industry will peak in 2025 under the ELS. In the BAU scenario, the larger energy-intensive industries will peak CO_2_ emissions in 2035. In addition, the CO_2_ emissions in pharmaceutical industry, automobile manufacturing, railway, ship, aerospace and other transportation equipment manufacturing, electrical machinery and equipment manufacturing, computer, communication and other electronic equipment manufacturing, instrumentation manufacturing, comprehensive utilization of waste resources, metal products, machinery and equipment repair industry will continue to increase during 2018 to 2050. In the ELS scenario, in order to achieve the CO_2_ emission peaking target in 2030, larger energy-intensive industries, such as chemical raw materials and chemical product manufacturing, ferrous metal smelting and rolling processing industry, non-metallic mineral products industry and food processing industry, are the main target industries for reducing CO_2_ emissions. However, the value added of these industries are estimated to account for more than 17% of total industrial value added in 2030. The compression of the development space of these industries will have a negative impact on Jilin’s economy. Previous study calculated by STIRPAT would peak the Jilin’s industrial CO_2_ emission in the same peak year under the optimal scenario with this study, which further illustrates the rationality of the industrial development predicted by this study [28]. The CO_2_ emission in optimal scenario decrease 29.94% and 22.63% compared with the BAU scenario in the previous study and this study. Due to the different calculation methods, the previous study did not give specific suggestions for reducing emissions in Jilin from the perspective of multiple industries and multiple energy types. This study provides specific insights into how each industry can reach peak CO_2_ emissions.

The industrial structure from the low rate of change to the medium rate of change in group have a significant CO_2_ emission reduction effect. However, the effect of CO_2_ emission reduction is obviously weakened from the medium rate group to the high rate group. This illustrates that with the increase of the change rate, the sensitivity of CO_2_ emission to the industrial structure decreases. The industrial structure optimized according to the original model is likely to lead to the bottleneck of CO_2_ emission reduction. The inhibiting effect of energy intensity of each industry on CO_2_ emission is second only to that of industrial structure, indicating that improving low-carbon technology is also very important for reducing CO_2_ emission. The vigorous development of clean coal technology and low-carbon technology can effectively reduce the peak of CO_2_ emission from industrial sector. The energy consumption types of each industry have little contribution to the peak value, and the change value is only −2.08 Mt in the ELS scenario. However, with the increase of the change rate, the inhibition effect on CO_2_ emission from the medium change rate group to the high change rate group was significantly higher than that from the low to the medium change rate group. In the future, the proportion of clean energy continues to increase, and the restraining effect of energy consumption types in various industries on CO_2_ emission will become more and more obvious. In addition, the results of previous studies indicated that China’s industrial energy-related CO_2_ emission and CO_2_ emission would peak 2025 and 2026 in the optimal scenario, separately [27,29]. In the ELS, the peak time in Jilin is slightly later than that in the above studies. Jilin should be the focus region for China to achieve the peak of CO_2_ emissions in 2030.

In the results section, the driving forces and mitigation potential of industrial CO_2_ emission during the period of 2018–2050 are introduced. To reduce CO_2_ emission peak of industrial sector, it can be reference to the development model of economy, technology, in the ELS. The suggestions are as follows: First, on the premise of stabilizing economic development, the growth rate of industry should be limited, especially the heavy industry. Energy-intensive industries are projected to peak CO_2_ emissions by 2025. At the same time, the development of tertiary industry is the inevitable choice of industrial optimization and transformation in Jilin. Second, adjust and optimize the industrial structure, vigorously develop emerging industries with low energy consumption and high added value, introduce more low-carbon industries, and close down backward production capacity. Third, vigorously develop clean coal technology and low-carbon technology to reduce energy intensity. In addition, due to the limited impact on industrial energy consumption types, certain measures can be taken, but the effect is not as obvious as the first three measures.

## 6. Conclusions

This study forecasts CO_2_ emission peak of industrial sector from multi-industry and multi-energy type perspectives. We incorporate 41 industries and 16 types of energy into the LEAP model in a typical heavy industrial region. This study provides targeted emission reduction suggestions for 41 industries. The results indicate:

(1) The peak times in the BAU, the ESS, the ELS, and the LCS, are 2040, 2040, 2030 and 2020 respectively, and the corresponding peak values are 165.65 Mt, 156.80 Mt, 128.16 Mt, and 114.17 Mt.
(2) ELS is the optimal scenario to coordinate the conflict between CO_2_ emission reduction and economic development with peak value 156.80 Mt in 2030.
(3) Under the ELS, the CO_2_ emissions of ferrous metal smelting and rolling processing industry, chemical raw materials and chemical products manufacturing, non-metallic mineral products industry, electric power and heat production and supply industry will experience a small increase (about 5%) during the 14th Five-Year Plan period, and will peak in 2025.
(4) Taking the ELS as an example, the influence degree of the four factors is as follows: industrial value added (1.27) > industrial structure (1.19) > energy intensity of each industry (1.12) > energy consumption types of each industry (1.02).

In order to achieve a lower peak of industrial sector CO_2_ emissions earlier, the following recommendations should be executed according to the results of the study. First, the development of heavy industry should be reasonably planned and limited. Secondly, upgrade the industrial structure and increase the proportion of emerging industries with low energy consumption and high benefit in the industrial value added. Third, promote the application of new energy-saving technologies to reduce the energy intensity of the industry. Finally, improve the energy consumption structure, reduce the use of coal, vigorously promote the use of renewable energy, and increase the proportion of clean energy.

However, this paper also has some deficiencies in the research: Since the statistical data of the terminal energy consumption of industrial sectors in Jilin has only appeared in the Statistical Yearbook of Jilin Province since 2011, the simulation forecast in this paper only includes the panel data from 2011 to 2020, which will affect the accuracy of the prediction. Future research will continue to explore the differences between industrial CO_2_ emissions in heavy and light industrial regions, from the perspective of multi-industry and multi-energy types.

## Figures and Tables

**Table 1:** Studies on predicting the industrial CO_2_ emission.

Category	References	Study Region	Issues Addressed	Method	Scenario
Studies on CO_2_ emissions in the holistic industrial sector	[29]	China	How to achieve the 2030 CO_2_ emission-reduction targets for China’s industrial sector	Extended LMDI model	BAU (the most possible to occur); N1 (higher efficiency improvement and stronger structural adjustment); N2 (higher efficiency improvement and weaker structural adjustment); N3 (lower efficiency improvement and stronger structural adjustment); N4 (lower efficiency improvement and weaker structural adjustment)
	[28]	Jilin Province	Peaking Industrial Energy-Related CO_2_ Emissions in Typical Transformation Region: Paths and Mechanism	STIRPAT model	Baseline scenario (BAU); energy-saving scenario (ESS); energy-saving and low-carbon scenario (ELS); low-carbon scenario (LCS)
	[30]	Henan Province	Exploring the Driving Forces and Reduction Potential of Industrial Energy-Related CO_2_ Emissions during 2001–2030	LMDI decomposition	Business as Usual (BAU); Efficiency Improvement (EI); Structural Optimization (SO); R&D Input (RD); Comprehensive Policy (CP) scenarios
Studies on CO_2_ emissions by industries in the industrial sector	[31]	China	Multi-scenario simulation on reducing CO_2_ emissions from China’s major manufacturing industries targeting 2060	based on China’s GHG inventory and uses Tier 2 of the IPCC	national determined contribution (NDC); carbon mitigation scenario (CMS); deep mitigation scenario (DMS)
	[32]	China	Peak energy consumption and CO_2_ emissions in China’s industrial sector	Modified GCAM	Reference (REF) scenario; Low-carbon (LC) scenario
	[33]	Thailand	A quantitative analysis of Low Carbon Society (LCS) measures in Thai industrial sector	AIM/Enduse model	Business As Usual (BAU); LCS scenarios; emission tax scenarios and reduction target scenarios
	[34]	European Union	Prospective scenarios on energy efficiency and CO_2_ emissions in the European Iron & Steel industry	Energy and CO_2_ simulation model	Baseline scenario (BS); two alternative scenarios (AS1 and AS2) to study the sensitivity of fuel and resource prices and CO_2_ emission prices
	[27]	China	Socioeconomic impact assessment of China’s CO_2_ emissions peak prior to 2030	an optimization model based on the input-output model	Peak in 2026; Peak in 2027; Peak in 2028; Peak in 2029; Peak in 2030

**Table 2:** Correlation coefficient of fossil energy.

Energy	Average Low Calorific Value (kJ/kg)	Carbon Content per Unit Calorific Value (tC/TJ)	Carbon Oxidation Rate (%)
	*NCV_j_*	*CC_j_*	*O_j_*
Raw coal	20,908	26.37	0.94
Washed coal	26,344	25.41	0.94
Coal products	10,454	25.8	0.94
Coke	28,435	29.5	0.93
Coke oven gas	17,355	12.1	0.98
Crude oil	41,816	20.1	0.98
Gasoline	43,070	18.9	0.98
Kerosene	43,070	19.6	0.98
Diesel	42,652	20.2	0.98
Fuel oil	41,816	21.1	0.98
Liquefied petroleum gas	50,179	17.2	0.98
Refinery dry gas	46,055	15.7	0.98
Other petroleum products	40,200	20	0.98
Natural gas	38,931	15.3	0.99

Note: Correlation coefficient of the above 14 types of fossil energy are sourced from Guidelines for Compiling Provincial Greenhouse Gas Inventories (NDRC [2011] No. 1041) [54], China Energy Statistical Yearbook 2020 [55] and General Principles for Calculation of Comprehensive Energy Consumption (GB/T 2589-2020) [56].

**Table 3:** The options of scenario’s parameters.

Scenarios	Industrial Value Added	Industrial Structure	Energy Intensity	Energy Consumption Types
LCS	Low	High	High	High
ELS	Medium	Medium	Medium	High
ESS	High	Low	Medium	Medium
BAU	High	Low	Low	Low

Note: high, medium and low represent high mode, medium mode and low mode respectively. According to the combination of high, medium and low modes of the four influencing factors, four scenarios of BAU, ESS, ELS and LCS is obtained.

**Table 4:** Peak prediction in 48 sub scenarios.

Factor	Rate	LCS		ELS		ESS		BAU	
		Year	Peak (Mt)	Year	Peak (Mt)	Year	Peak (Mt)	Year	Peak (Mt)
Industrial value added	Low	2020	114.17	2021	114.85	2025	118.09	2025	121.68
	Medium	2023	118.22	2030	128.15	2030	136.7	2030	142.82
	High	2035	127.23	2040	145.55	2040	156.8	2040	165.65
Industrial structure	Low	2024	123.7	2030	142.36	2040	156.8	2040	165.65
	Medium	2020	117.7	2030	128.15	2040	138.23	2040	144.94
	High	2020	114.17	2030	119.36	2035	126.53	2040	131.44
Energy intensity	Low	2020	118.89	2030	136.04	2040	159.02	2040	165.65
	Medium	2020	116.45	2030	128.15	2040	148.5	2040	155.72
	High	2020	114.17	2028	121.04	2035	137.62	2035	141.18
Energy consumption types	Low	2021	115.31	2030	130.24	2040	157.27	2040	165.65
	Medium	2021	115.13	2030	130.09	2040	156.8	2040	158.37
	High	2020	114.17	2030	128.15	2040	145.68	2035	147.82

## Appendix A

**Table A1:** Scenario setting.

Scenarios	Parameter Descriptions	References
BAU	The specific manifestations of the BAU are rapid industrial development, rapid growth of industrial value added, traditional industries with high energy dependence account for a large proportion of industrial added value, high energy intensity, limited energy efficiency level, and coal and oil in the final energy consumption. The proportion of other fossil energy sources is relatively high. Among them, the industrial added value increased steadily at 6% in accordance with the 13th Five-Year Development Plan of Jilin Province, and the energy intensity and energy consumption type remained at the current level. In 2030, the proportion of transportation equipment manufacturing industry will be 32.14%; the proportion of raw coal in the agricultural and sideline food processing industry will reach 81.18%, and the proportion of non-fossil energy consumption in the industrial sector will increase to more than 15%	Jilin Province Action Plan for Promoting Steady Growth of Industrial Economy [59]. The 13th Five-Year Development Plan for Industry of Jilin Province [57]. The 14th Five-Year Development Plan for Industry of Jilin Province [60]. The 14th Five-Year Plan for the National Economy of Jilin Province [61].
ESS	The growth rate of industrial value added and the state of industrial structure are basically the same as those in the BAU scenario. Compared with the base year, the energy intensity will be reduced by 14.6–52.5% in multiple industries by 2050; the proportion of fossil energy will be decreased to 6.17–95.28% by industries. Compared with BAU, it will further improve energy utilization efficiency and promote the application of energy-saving technologies.	Natural Gas Utilization Plan of Jilin Province (2016–2025) [62]. Jilin Province Action Plan for Promoting Steady Growth of Industrial Economy [59]. The 14th Five-Year Development Plan for Industry of Jilin Province [60]. The 14th Five-Year Plan for the National Economy of Jilin Province [61]. The 13th Five-Year Development Plan for Industry of Jilin Province [57].
ELS	Based on the state in the ESS, further promote the use of clean energy, vigorously develop industries with low energy consumption and high added value, and reduce the proportion of industrial added value in traditional energy-dependent industries. The proportion of fossil energy will further be reduce to 4.62–95.14% by different industries. By 2050, the proportion of transportation equipment manufacturing industry will increase to 34.53%, the proportion of communication equipment, computer and other electronic equipment manufacturing industry will increase to 0.72%, and the proportion of ferrous metal mining and dressing industry will decrease to 0.37%.	The 14th Five-Year Plan for the Development of the Petrochemical Industry in Jilin Province [63]. Jilin Province Action Plan for Promoting Steady Growth of Industrial Economy [59]. The 14th Five-Year Development Plan for Industry of Jilin Province [60]. The 14th Five-Year Plan for the Development of Metallurgical Building Materials Industry in Jilin Province [64]. The 14th Five-Year Industrial Green Development Plan [65].
LCS	The growth rate of industrial value added will further decrease. Compared with the base year, the energy intensity of various industries in the industrial sector will drop by 24.97%–65.47% by 2050; the proportion of industries with low pollution and high added value will continue to increase; the energy structure will be further adjusted, and the proportion of clean energy will be basically stable at a relatively high level. By 2050, the proportion of ferrous metal smelting and rolling processing industry will decrease from 4.59% in the base year to 0.46%; the proportion of chemical raw materials and chemical products manufacturing industry will decrease to 1.55%.	New Energy Vehicle Industry Development Plan (2021–2035) [66]. The 14th Five-Year Plan for Scientific and Technological Innovation in the Energy Sector [67]. Jilin Province Action Plan for Promoting Steady Growth of Industrial Economy [59]. The 14th Five-Year Development Plan for Industry of Jilin Province [60]. The 14th Five-Year Industrial Green Development Plan [65].

## Appendix B. Industrial Value Added

The industrial value added of Jilin Province increased steadily from 2005 to 2018, but the growth rate of industrial value added showed a downward trend. According to the 14th Five-Year Plan of Jilin Province, the industrial value added would grow at no less than 6% per year, as a reference for the initial stage of industrial value added of Jilin Province in the high mode. However, it is unrealistic for Jilin Province to maintain long-term economic growth of more than 6% in the future. Therefore, the growth rate of the industrial value added of Jilin Province in the high mode is 6%. The growth rate in the medium mode and the low mode has a certain degree of reduction. The parameter setting is shown in Appendix B.

**Table A2:** Parameter setting of industrial value added in Jilin Province (100 million yuan).

Mode	2018	2020	2025	2030	2035	2040	2045	2050
High Mode	6794	7160	9022	10,736	11,917	12,513	12,263	11,895
Medium Mode	6794	7000	8610	9902	10,496	10,391	10,183	9776
Low Mode	6794	6840	7866	8180	8017	7696	7234	6656

## Appendix C. Industrial Structure

The proportion of the value added of each industry in the industrial value added represents the industrial structure. At present, the pillar industries of Jilin are still automobile, petrochemical and agricultural product processing industry. According to the 14th Five-Year Plan of Jilin Province, the industrial value added of Jilin should grow at an annual rate of no less than 6%. Across industries, if the growth rate of value added of the industry is higher than that of the complete industrial value added, the proportion of this industry in the industrial sector will increase accordingly. However, it is impossible for the value added of each industry to maintain a sustained high-speed growth and the growth rate is bound to decrease. Based on this, the model parameters of the industrial structure are set as the low mode. The specific parameter setting in medium mode and high mode is shown in Appendix C (taking the proportion of each industry in the input-output table of Jilin Province in 2012 as the base year data).

**Table A3:** Change of industrial structure in industrial sectors.

Industries	Low Mode							Medium Mode							High Mode						
	2020	2025	2030	2035	2040	2045	2050	2020	2025	2030	2035	2040	2045	2050	2020	2025	2030	2035	2040	2045	2050
Coal mining and washing industry	−5.4%	−25%	−36%	−45%	−54%	−61%	−67%	−4.9%	−24%	−35%	−45%	−54%	−62%	−68%	−4.3%	−22%	−34%	−44%	−54%	−63%	−70%
Ferrous metal mining and dressing industry	−5.4%	−25%	−36%	−45%	−54%	−61%	−67%	−4.9%	−24%	−35%	−45%	−54%	−62%	−68%	−4.3%	−22%	−34%	−44%	−54%	−63%	−70%
Non−ferrous metal mining and dressing industry	−5.4%	−25%	−36%	−45%	−54%	−61%	−67%	−4.9%	−24%	−35%	−45%	−54%	−62%	−68%	−4.3%	−22%	−34%	−44%	−54%	−63%	−70%
Mining ancillary activities	−5.4%	−25%	−36%	−45%	−54%	−61%	−67%	−4.9%	−24%	−35%	−45%	−54%	−62%	−68%	−4.3%	−22%	−34%	−44%	−54%	−63%	−70%
Nonmetallic Mining and Dressing Industry	−5.4%	−25%	−36%	−45%	−54%	−61%	−67%	−4.9%	−24%	−35%	−45%	−54%	−62%	−68%	−4.3%	−22%	−34%	−44%	−54%	−63%	−70%
Oil and Gas Extraction Industry	−5.4%	−25%	−36%	−45%	−54%	−61%	−67%	−4.9%	−24%	−35%	−45%	−54%	−62%	−68%	−4.3%	−22%	−34%	−44%	−54%	−63%	−70%
Other mining industry	0.0%	−1%	−2%	−3%	−5%	−7%	−10%	0.5%	1%	0%	−3%	−6%	−16%	−13%	1.2%	3%	2%	−1%	−5%	−13%	−18%
Transportation Equipment Manufacturing	3.0%	14%	20%	24%	26%	27%	26%	3.6%	17%	23%	28%	30%	30%	29%	4.3%	20%	26%	31%	33%	29%	28%
Agricultural and sideline food processing industry	0.0%	−1%	−2%	−3%	−5%	−7%	−10%	0.5%	1%	0%	−3%	−6%	−9%	−13%	1.2%	3%	2%	−1%	−5%	−13%	−18%
Chemical raw materials and chemical products manufacturing	−3.6%	−18%	−26%	−34%	−41%	−48%	−54%	−8.7%	−39%	−51%	−61%	−66%	−70%	−74%	−11.4%	−50%	−64%	−74%	−81%	−86%	−90%
Non-metallic mineral products industry	1.6%	7%	9%	10%	10%	9%	7%	2.1%	9%	11%	13%	13%	12%	9%	3.2%	14%	16%	17%	16%	9%	5%
Wine, Beverage and Refined Tea Manufacturing	0.0%	−1%	−2%	−3%	−5%	−7%	−10%	0.5%	1%	0%	−3%	−6%	−9%	−13%	−8.1%	−38%	−50%	−61%	−68%	−13%	−18%
Ferrous metal smelting and rolling industry	−5.3%	−25%	−36%	−45%	−54%	−61%	−67%	−8.7%	−39%	−51%	−61%	−69%	−74%	−79%	−11.4%	−50%	−64%	−74%	−82%	−87%	−90%
Wood processing and wood, bamboo, rattan, palm and grass products industry	−3.6%	−18%	−26%	−34%	−41%	−48%	−54%	−3.2%	−16%	−25%	−34%	−41%	−49%	−55%	−2.6%	−14%	−23%	−32%	−41%	−51%	−58%
Petroleum processing, coking and nuclear fuel processing industries	−3.6%	−18%	−26%	−34%	−41%	−48%	−54%	−3.2%	−16%	−25%	−34%	−41%	−49%	−55%	−2.6%	−14%	−23%	−32%	−41%	−51%	−58%
Special equipment manufacturing	3.9%	20%	30%	41%	51%	62%	73%	7.0%	36%	55%	76%	97%	115%	134%	12.6%	69%	102%	139%	179%	211%	241%
food manufacturing	0.0%	−1%	−2%	−3%	−5%	−7%	−10%	0.5%	1%	0%	−3%	−6%	−9%	−13%	1.2%	3%	2%	−1%	−5%	−13%	−18%
Pharmaceutical Manufacturing	12.7%	72%	124%	191%	276%	383%	517%	13.3%	75%	127%	193%	273%	374%	500%	14.2%	79%	133%	198%	276%	355%	466%
General equipment manufacturing	3.9%	20%	30%	41%	51%	62%	73%	7.0%	36%	55%	76%	87%	98%	109%	12.6%	69%	102%	139%	179%	211%	241%
Electrical machinery and equipment manufacturing	3.9%	20%	30%	41%	51%	62%	73%	4.5%	22%	32%	41%	59%	73%	83%	5.2%	25%	35%	44%	60%	66%	73%
metal products industry	3.9%	20%	30%	41%	51%	62%	73%	4.5%	22%	32%	41%	50%	59%	68%	5.2%	25%	35%	44%	52%	53%	59%
Chemical fiber manufacturing	0.0%	−1%	−2%	−3%	−5%	−7%	−10%	0.5%	1%	0%	−3%	−6%	−9%	−13%	1.2%	3%	2%	−1%	−5%	−13%	−18%
Textile and apparel industry	0.0%	−1%	−2%	−3%	−5%	−7%	−10%	0.5%	1%	0%	−3%	−6%	−9%	−13%	1.2%	3%	2%	−1%	−5%	−13%	−18%
Paper and paper products industry	−3.6%	−18%	−26%	−34%	−41%	−48%	−54%	−3.2%	−16%	−25%	−34%	−41%	−49%	−55%	−2.6%	−14%	−23%	−32%	−41%	−51%	−58%
textile industry	0.0%	−1%	−2%	−3%	−5%	−7%	−10%	0.5%	1%	0%	−3%	−6%	−9%	−13%	1.2%	3%	2%	−1%	−5%	−13%	−18%
Communication equipment, computer and other electronic equipment manufacturing	6.0%	31%	49%	69%	91%	114%	139%	6.6%	34%	51%	70%	89%	110%	132%	7.3%	37%	55%	73%	91%	101%	119%
furniture manufacturing	0.0%	−1%	−2%	−3%	−5%	−7%	−10%	0.5%	1%	0%	−3%	−6%	−9%	−13%	1.2%	3%	2%	−1%	−5%	−13%	−18%
Printing and recording media reproduction industry	0.0%	−1%	−2%	−3%	−5%	−7%	−10%	0.5%	1%	0%	−3%	−6%	−9%	−13%	1.2%	3%	2%	−1%	−5%	−13%	−18%
Non-ferrous metal smelting and calendaring industry	−5.3%	−25%	−36%	−45%	−54%	−61%	−67%	−4.9%	−24%	−35%	−45%	−54%	−62%	−68%	−4.3%	−22%	−34%	−44%	−54%	−63%	−70%
Rubber and plastic products industry	0.0%	−1%	−2%	−3%	−5%	−7%	−10%	0.5%	1%	0%	−3%	−6%	−9%	−13%	1.2%	3%	2%	−1%	−5%	−13%	−18%
Instrumentation Manufacturing	3.9%	20%	30%	41%	51%	62%	73%	4.5%	22%	32%	41%	50%	59%	68%	5.2%	25%	35%	44%	52%	53%	59%
Tobacco Products Industry	0.0%	−1%	−2%	−3%	−5%	−7%	−10%	0.5%	1%	0%	−3%	−6%	−9%	−13%	1.2%	3%	2%	−1%	−5%	−13%	−18%
Cultural and educational, industrial beauty, sports and entertainment products manufacturing	0.0%	−1%	−2%	−3%	−5%	−7%	−10%	0.5%	1%	0%	−3%	−6%	−9%	−13%	1.2%	3%	2%	−1%	−5%	−13%	−18%
Other manufacturing	0.0%	−1%	−2%	−3%	−5%	−7%	−10%	0.5%	1%	0%	−3%	−6%	−9%	−13%	1.2%	3%	2%	−1%	−5%	−13%	−18%
Leather, fur, feathers and their products and footwear	0.0%	−1%	−2%	−3%	−5%	−7%	−10%	0.5%	1%	0%	−3%	−6%	−9%	−13%	1.2%	3%	2%	−1%	−5%	−13%	−18%
Comprehensive utilization of waste resources	0.0%	−1%	−2%	−3%	−5%	−7%	−10%	0.5%	1%	0%	−3%	−6%	−9%	−13%	1.2%	3%	2%	−1%	−5%	−13%	−18%
Repair of metal products, machinery and equipment	0.0%	−1%	−2%	−3%	−5%	−7%	−10%	0.5%	1%	0%	−3%	−6%	−9%	−13%	1.2%	3%	2%	−1%	−5%	−13%	−18%
Water production and supply industry	0.0%	−1%	−2%	−3%	−5%	−7%	−10%	0.5%	1%	0%	−3%	−6%	−9%	−13%	1.2%	3%	2%	−1%	−5%	−13%	−18%
Electricity and heat production and supply industry	1.2%	5%	9%	6%	5%	3%	0%	1.7%	7%	8%	8%	7%	5%	3%	2.8%	12%	14%	15%	13%	7%	3%
Gas production and supply industry	0.0%	−1%	1%	−3%	−5%	−7%	−10%	0.5%	1%	0%	−3%	−6%	−9%	−13%	1.2%	3%	2%	−1%	−5%	−13%	−18%

## Appendix D. Energy Intensity of Various Industries

During 2010–2017, the energy intensity of terminal energy consumption in Jilin province showed a gradually decreasing trend. Since there is no specific numerical regulation on the development of energy intensity of each industry in the existing plans or policies of Jilin Province, this paper uses the rate of change to predict the change of energy intensity of each industry sector in the low, medium and high three rate modes. The energy intensity of each industry during 2010–2017 is used as panel data to analyze the change rate of energy intensity in each year during 2010–2017. Based on the average rate of change, this study sets reducing rate of the energy intensity of each industry in the forecast year. See Appendix C for the change rates of energy intensity and specific parameter settings of major industries in Jilin Province during 2010–2017.

**Table A4:** Change of energy intensity in industrial sectors of Jilin Province.

Energy Intensity	High Mode		Medium Mode		Low Mode	
	2030	2050	2030	2050	2030	2050
Coal mining and washing industry	−38.62%	−56.08%	−34.18%	−48.67%	−24.68%	−31.97%
Oil and Gas Extraction Industry	−38.62%	−56.08%	−31.89%	−44.58%	−24.68%	−31.97%
Ferrous metal mining and dressing industry	−16.95%	−24.97%	−11.54%	−14.60%	−7.32%	−10.52%
Non-ferrous metal mining and dressing industry	−24.65%	−39.93%	−19.57%	−30.27%	−14.27%	−19.32%
Nonmetallic Mining and Dressing Industry	−34.18%	−48.67%	−29.54%	−40.22%	−24.68%	−31.63%
Agricultural and sideline food processing industry	−34.18%	−48.67%	−29.54%	−40.22%	−24.68%	−31.63%
food manufacturing	−44.90%	−65.47%	−36.43%	−52.50%	−27.14%	−35.58%
Wine, Beverage and Refined Tea Manufacturing	−24.65%	−39.93%	−19.57%	−30.27%	−14.27%	−19.32%
textile industry	−34.18%	−48.67%	−29.54%	−40.22%	−24.68%	−31.63%
Wood processing and wood, bamboo, rattan, palm and grass products industry	−34.18%	−48.67%	−29.54%	−40.22%	−24.68%	−31.63%
Paper and paper products industry	−34.18%	−48.67%	−29.54%	−40.22%	−24.68%	−31.63%
Printing and recording media reproduction industry	−16.95%	−24.97%	−11.54%	−14.60%	−7.32%	−10.52%
Petroleum processing, coking and nuclear fuel processing industries	−16.95%	−24.97%	−11.54%	−14.60%	−7.32%	−10.52%
Chemical raw materials and chemical products manufacturing	−16.95%	−24.97%	−11.54%	−14.60%	−7.32%	−10.52%
Pharmaceutical Manufacturing	−16.95%	−24.97%	−11.54%	−14.60%	−7.32%	−10.52%
Chemical fiber manufacturing	−24.65%	−39.93%	−19.57%	−30.27%	−14.27%	−19.32%
Rubber and plastic products industry	−16.95%	−24.97%	−11.54%	−14.60%	−7.32%	−10.52%
Non-metallic mineral products industry	−34.18%	−48.67%	−29.54%	−40.22%	−24.68%	−31.63%
Ferrous metal smelting and rolling industry	−24.65%	−39.93%	−19.57%	−30.27%	−14.27%	−19.32%
Non-ferrous metal smelting and calendaring industry	−44.90%	−65.47%	−36.43%	−52.50%	−27.14%	−35.56%
metal products industry	−34.18%	−48.67%	−29.54%	−40.22%	−24.68%	−31.63%
General equipment manufacturing	−24.65%	−39.93%	−19.57%	−30.27%	−14.27%	−19.32%
Special equipment manufacturing	−16.95%	−24.97%	−11.54%	−14.60%	−7.32%	−10.52%
Transportation Equipment Manufacturing	−16.95%	−24.97%	−11.54%	−14.60%	−7.32%	−10.52%
Electrical machinery and equipment manufacturing	−16.95%	−24.97%	−11.54%	−14.60%	−7.32%	−10.52%
Communication equipment, computer and other electronic equipment manufacturing	−16.95%	−24.97%	−11.54%	−14.60%	−7.32%	−10.52%
Electricity and heat production and supply industry	−34.18%	−48.67%	−29.54%	−40.22%	−24.68%	−31.63%
Water production and supply industry	−16.95%	−24.97%	−11.54%	−18.55%	−7.32%	−10.52%

## Appendix E. Energy Consumption Types of Diverse Industries

The energy consumption types varies extremely between industries. The 14th Five-Year Plan of Energy and the 14th Five-Year Plan of environmental Protection of Jilin Province propose to reduce the consumption of coal and increase the proportion of non-fossil energy in the terminal energy consumption. However, there is no policy direction that clearly defines the energy consumption types of each industry sector. This paper analyzes the energy consumption types of each industry and the change rate and change trend according to the change of energy consumption ratio of each industry in Jilin Province during 2010–2017. Then, this study sets the parameter of energy consumption types change of each industry in Jilin Province in low, medium and high three modes on this basis. The proportion of fossil energy in final energy consumption is shown in Appendix E. Due to the huge amount of data, the agricultural and sideline food processing industry, petroleum processing industry, coking and nuclear fuel processing industry, and chemical raw material and chemical product manufacturing industry are selected as examples to set the energy consumption type parameters. The specific parameters are shown in Appendix E.

**Table A5:** Setting the proportion of fossil energy.

Industries	Low Mode		Medium Mode		High Mode	
	2030	2050	2030	2050	2030	2050
Coal mining and washing industry	65.05%	62.77%	62.07%	59.54%	58.25%	52.54%
Oil and Gas Extraction Industry	75.19%	76.79%	76.12%	79.54%	78.10%	81.91%
Ferrous metal mining and dressing industry	71.31%	71.89%	69.40%	69.17%	68.59%	67.16%
Non-ferrous metal mining and dressing industry	69.71%	73.81%	65.86%	68.15%	62.16%	63.15%
Agricultural and sideline food processing industry	90.71%	91.99%	87.78%	90.80%	83.84%	85.32%
food manufacturing	73.67%	70.73%	70.75%	66.00%	67.99%	62.60%
Wine, Beverage and Refined Tea Manufacturing	48.69%	44.17%	45.77%	38.38%	42.97%	33.28%
textile industry	56.64%	51.24%	53.15%	44.31%	49.80%	38.22%
Wood processing and wood, bamboo, rattan, palm and grass products industry	89.18%	86.29%	87.02%	82.94%	84.63%	80.88%
Paper and paper products industry	79.26%	77.36%	76.58%	74.98%	73.01%	68.43%
Petroleum processing, coking and nuclear fuel processing industries	80.31%	83.28%	83.50%	87.79%	87.01%	92.44%
Chemical raw materials and chemical products manufacturing	58.17%	56.85%	58.87%	59.40%	59.12%	59.53%
Pharmaceutical Manufacturing	87.51%	85.27%	85.20%	81.34%	82.54%	80.07%
Chemical fiber manufacturing	8.24%	8.61%	8.72%	9.78%	9.05%	10.15%
Rubber and plastic products industry	70.04%	69.85%	71.81%	72.36%	73.95%	75.31%
Non-metallic mineral products industry	89.14%	90.15%	87.97%	89.38%	86.54%	88.60%
Ferrous metal smelting and rolling industry	94.82%	95.23%	95.46%	95.58%	95.95%	95.14%
Non-ferrous metal smelting and calendaring industry	62.59%	58.70%	62.11%	57.41%	61.99%	55.44%
metal products industry	54.28%	52.06%	54.69%	54.54%	54.20%	51.32%
General equipment manufacturing	81.03%	79.05%	79.85%	77.31%	78.12%	76.09%
Special equipment manufacturing	72.47%	71.45%	71.06%	71.36%	68.65%	66.40%
Transportation Equipment Manufacturing	42.17%	40.66%	41.64%	39.03%	41.48%	37.78%
Electrical machinery and equipment manufacturing	90.33%	90.19%	91.90%	95.28%	92.44%	94.68%
Communication equipment, computer and other electronic equipment manufacturing	3.35%	2.85%	3.13%	2.44%	2.92%	2.09%
Electricity and heat production and supply industry	60.16%	58.09%	57.44%	55.18%	53.94%	48.76%
Water production and supply industry	8.47%	7.19%	7.91%	6.17%	7.23%	4.62%

**Table A6:** Setting energy consumption types parameter of agricultural and sideline food processing industry.

Mode	Year	The Raw Coal	Washed Coal	Coal Products	Coke	Coke Oven Gas	Crude Oil	Gasoline	Kerosene	Diesel	Fuel Oil	LPG	Refinery Dry Gas	Other Petroleum Products	Natural Gas	The Thermal	Electric Power
Low rate mode	2020	84.96%	0.01%	0.02%	-	-	-	3.50%	0.01%	3.88%	-	-	-	-	0.17%	0.36%	6.14%
	2025	82.84%	0.01%	0.02%	-	-	-	3.95%	0.01%	4.39%	-	-	-	-	0.17%	0.36%	6.94%
	2030	81.18%	0.01%	0.02%	-	-	-	4.43%	0.01%	4.91%	-	-	-	-	0.17%	0.36%	7.78%
	2035	79.96%	0.01%	0.02%	-	-	-	4.91%	0.01%	5.45%	-	-	-	-	0.17%	0.36%	8.63%
	2040	79.16%	0.01%	0.02%	-	-	-	5.41%	0.01%	6.00%	-	-	-	-	0.17%	0.36%	9.50%
	2045	78.77%	0.01%	0.02%	-	-	-	5.89%	0.01%	6.54%	-	-	-	-	0.17%	0.36%	10.35%
	2050	78.37%	0.01%	0.02%	-	-	-	6.36%	0.01%	7.06%	-	-	-	-	0.17%	0.36%	11.18%
Medium rate mode	2020	83.21%	0.01%	0.02%	-	-	-	3.59%	0.01%	3.99%	-	-	-	-	0.17%	0.36%	6.31%
	2025	79.88%	0.01%	0.02%	-	-	-	4.16%	0.01%	4.62%	-	-	-	-	0.17%	0.36%	7.32%
	2030	77.48%	0.01%	0.02%	-	-	-	4.79%	0.01%	5.32%	-	-	-	-	0.17%	0.36%	8.41%
	2035	75.93%	0.01%	0.02%	-	-	-	5.46%	0.01%	6.06%	-	-	-	-	0.17%	0.36%	9.59%
	2040	75.17%	0.01%	0.02%	-	-	-	6.17%	0.01%	6.85%	-	-	-	-	0.17%	0.36%	10.84%
	2045	74.80%	0.01%	0.02%	-	-	-	6.91%	0.01%	7.67%	-	-	-	-	0.17%	0.36%	12.14%
	2050	74.42%	0.01%	0.02%	-	-	-	7.67%	0.01%	8.51%	-	-	-	-	0.17%	0.36%	13.47%
High rate mode	2020	81.46%	0.01%	0.02%	-	-	-	3.68%	0.01%	4.09%	-	-	-	-	0.17%	0.36%	6.47%
	2025	76.57%	0.01%	0.02%	-	-	-	4.38%	0.01%	4.86%	-	-	-	-	0.17%	0.36%	7.70%
	2030	72.74%	0.01%	0.02%	-	-	-	5.17%	0.01%	5.74%	-	-	-	-	0.17%	0.36%	9.08%
	2035	69.83%	0.01%	0.02%	-	-	-	6.05%	0.01%	6.72%	-	-	-	-	0.17%	0.36%	10.63%
	2040	67.74%	0.01%	0.02%	-	-	-	7.02%	0.01%	7.79%	-	-	-	-	0.17%	0.36%	12.33%
	2045	66.38%	0.01%	0.02%	-	-	-	8.07%	0.01%	8.96%	-	-	-	-	0.17%	0.36%	14.18%
	2050	65.72%	0.01%	0.02%	-	-	-	9.20%	0.01%	10.21%	-	-	-	-	0.17%	0.36%	16.16%

**Table A7:** Setting energy consumption types parameter for petroleum processing, coking and nuclear fuel processing industries.

Mode	Year	The Raw Coal	Washed Coal	Coal Products	Coke	Coke Oven Gas	Crude Oil	Gasoline	Kerosene	Diesel	Fuel Oil	LPG	Refinery Dry Gas	Other Petroleum Products	Natural Gas	The Thermal	Electric Power
Low rate mode	2020	8.28%	2.31%	-	-	0.88%	-	0.79%	-	3.06%	-	27.01%	15.29%	-	18.33%	0.69%	21.91%
	2025	7.79%	2.17%	-	-	0.88%	-	0.74%	-	3.15%	-	29.17%	14.37%	-	19.80%	0.69%	19.50%
	2030	7.40%	2.06%	-	-	0.88%	-	0.70%	-	3.22%	-	31.21%	13.65%	-	21.18%	0.69%	17.74%
	2035	7.10%	1.98%	-	-	0.88%	-	0.67%	-	3.28%	-	32.77%	13.10%	-	22.24%	0.69%	16.50%
	2040	6.89%	1.92%	-	-	0.88%	-	0.65%	-	3.31%	-	33.75%	12.71%	-	22.91%	0.69%	15.68%
	2045	6.75%	1.88%	-	-	0.88%	-	0.64%	-	3.35%	-	34.09%	12.46%	-	23.14%	0.69%	15.21%
	2050	6.68%	1.86%	-	-	0.88%	-	0.63%	-	3.36%	-	34.26%	12.33%	-	23.25%	0.69%	15.05%
Medium rate mode	2020	8.11%	2.26%	-	-	0.88%	-	0.77%	-	3.12%	-	27.75%	14.96%	-	18.83%	0.69%	21.40%
	2025	7.46%	2.08%	-	-	0.88%	-	0.71%	-	3.28%	-	30.80%	13.76%	-	20.91%	0.69%	18.62%
	2030	6.94%	1.93%	-	-	0.88%	-	0.66%	-	3.41%	-	33.89%	12.80%	-	23.00%	0.69%	16.57%
	2035	6.52%	1.82%	-	-	0.88%	-	0.62%	-	3.54%	-	36.60%	12.03%	-	24.84%	0.69%	15.08%
	2040	6.19%	1.73%	-	-	0.88%	-	0.59%	-	3.65%	-	38.43%	11.43%	-	26.08%	0.69%	14.03%
	2045	5.95%	1.66%	-	-	0.88%	-	0.56%	-	3.76%	-	38.43%	10.97%	-	26.08%	0.69%	13.32%
	2050	5.77%	1.61%	-	-	0.88%	-	0.55%	-	3.84%	-	38.43%	10.64%	-	26.08%	0.69%	12.92%
High rate mode	2020	7.93%	2.21%	-	-	0.88%	-	0.75%	-	3.18%	-	28.50%	14.63%	-	19.34%	0.69%	20.90%
	2025	7.14%	1.99%	-	-	0.88%	-	0.68%	-	3.40%	-	32.48%	13.17%	-	22.05%	0.69%	17.76%
	2030	6.49%	1.81%	-	-	0.88%	-	0.62%	-	3.61%	-	36.71%	11.98%	-	24.91%	0.69%	15.46%
	2035	5.97%	1.66%	-	-	0.88%	-	0.57%	-	3.82%	-	39.64%	11.02%	-	26.90%	0.69%	13.76%
	2040	5.56%	1.55%	-	-	0.88%	-	0.53%	-	4.02%	-	42.02%	10.25%	-	28.52%	0.69%	12.52%
	2045	5.22%	1.46%	-	-	0.88%	-	0.50%	-	4.22%	-	42.44%	9.64%	-	28.80%	0.69%	11.64%
	2050	4.96%	1.38%	-	-	0.88%	-	0.47%	-	4.34%	-	42.44%	9.15%	-	28.80%	0.69%	11.06%

**Table A8:** Setting energy consumption types parameter for manufacturing of chemical raw materials and chemical products.

Mode	Year	The Raw Coal	Washed Coal	Coal Products	Coke	Coke Oven Gas	Crude Oil	Gasoline	Kerosene	Diesel	Fuel Oil	LPG	Refinery Dry Gas	Other Petroleum Products	Natural Gas	The Thermal	Electric Power
Low rate mode	2020	11.59%	2.51%	0.01%	-	-	0.28%	0.41%	-	0.60%	4.63%	-	6.88%	26.65%	7.26%	31.37%	5.89%
	2025	10.32%	2.36%	0.01%	-	-	0.28%	0.41%	-	0.60%	4.35%	-	7.09%	27.45%	6.47%	32.32%	5.53%
	2030	9.39%	2.24%	0.01%	-	-	0.28%	0.41%	-	0.60%	4.13%	-	7.23%	28.00%	5.88%	32.96%	5.26%
	2035	8.73%	2.15%	0.01%	-	-	0.28%	0.41%	-	0.60%	3.97%	-	7.38%	28.56%	5.47%	33.62%	5.05%
	2040	8.29%	2.09%	0.01%	-	-	0.28%	0.41%	-	0.60%	3.85%	-	7.45%	28.85%	5.20%	33.96%	4.90%
	2045	8.05%	2.05%	0.01%	-	-	0.28%	0.41%	-	0.60%	3.77%	-	7.52%	29.14%	5.04%	34.30%	4.80%
	2050	7.96%	2.03%	0.01%	-	-	0.28%	0.41%	-	0.60%	3.73%	-	7.56%	29.28%	4.99%	34.47%	4.75%
Medium rate mode	2020	11.32%	2.46%	0.01%	-	-	0.28%	0.41%	-	0.60%	4.53%	-	7.02%	27.17%	7.10%	31.98%	5.76%
	2025	9.85%	2.26%	0.01%	-	-	0.28%	0.41%	-	0.60%	4.16%	-	7.37%	28.53%	6.18%	33.58%	5.30%
	2030	8.77%	2.10%	0.01%	-	-	0.28%	0.41%	-	0.60%	3.87%	-	7.66%	29.67%	5.50%	34.93%	4.93%
	2035	7.98%	1.98%	0.01%	-	-	0.28%	0.41%	-	0.60%	3.64%	-	7.97%	30.86%	5.00%	36.32%	4.63%
	2040	7.42%	1.88%	0.01%	-	-	0.28%	0.41%	-	0.60%	3.46%	-	8.21%	31.78%	4.65%	37.41%	4.40%
	2045	7.05%	1.80%	0.01%	-	-	0.28%	0.41%	-	0.60%	3.32%	-	8.45%	32.74%	4.42%	38.53%	4.23%
	2050	6.84%	1.75%	0.01%	-	-	0.28%	0.41%	-	0.60%	3.22%	-	8.62%	33.39%	4.29%	39.31%	4.10%
High rate mode	2020	11.06%	2.40%	0.01%	-	-	0.28%	0.41%	-	0.60%	4.43%	-	7.15%	27.69%	6.93%	32.59%	5.63%
	2025	9.40%	2.16%	0.01%	-	-	0.28%	0.41%	-	0.60%	3.98%	-	7.65%	29.35%	5.89%	34.55%	5.07%
	2030	8.18%	1.97%	0.01%	-	-	0.28%	0.41%	-	0.60%	3.63%	-	8.11%	30.82%	5.13%	36.28%	4.61%
	2035	7.28%	1.81%	0.01%	-	-	0.28%	0.41%	-	0.60%	3.34%	-	8.60%	32.05%	4.56%	37.73%	4.24%
	2040	6.62%	1.68%	0.01%	-	-	0.28%	0.41%	-	0.60%	3.10%	-	9.03%	33.01%	4.15%	38.86%	3.95%
	2045	6.16%	1.58%	0.01%	-	-	0.28%	0.41%	-	0.60%	2.92%	-	9.48%	34.00%	3.86%	40.02%	3.71%
	2050	5.85%	1.50%	0.01%	-	-	0.28%	0.41%	-	0.60%	2.77%	-	9.76%	34.68%	3.67%	40.82%	3.53%

## Appendix F. Industry Code Comparison Table

**Table A9:** The full names of industries.

Industry Code	Industry Name
MWC	Coal mining and washing industry
EPN	Oil and Gas Extraction Industry
MPF	Ferrous metal mining and dressing industry
PNM	Non-ferrous metal mining and dressing industry
PNO	Nonmetallic Mining and Dressing Industry
PSA	Mining Professional and Auxiliary Activities
MOO	Other mining industry
PFA	Agricultural and sideline food processing industry
MFO	food manufacturing
MLB	Wine, Beverage and Refined Tea Manufacturing
MTO	Tobacco Products Industry
MTE	textile industry
MTW	Textile and apparel industry
MLF	Leather, fur, feathers and their products and footwear
PTM	Wood processing and wood, bamboo, rattan, palm and grass products industry
MFU	furniture manufacturing
MPP	Paper and paper products industry
PRR	Printing and recording media reproduction industry
MAC	Cultural and educational, industrial beauty, sports and entertainment products manufacturing
PPC	Petroleum, coal and other fuel processing industries
MRC	Chemical raw materials and chemical products manufacturing
MME	Pharmaceutical Manufacturing
MCF	Chemical fiber manufacturing
MPR	Rubber and plastic products industry
MNM	Non-metallic mineral products industry
SPF	Ferrous metal smelting and rolling industry
SPN	Non-ferrous metal smelting and calendering industry
MMP	metal products industry
MGP	General equipment manufacturing
MSP	Special equipment manufacturing
MAU	Automotive Manufacturing
MRS	Railway, marine, aerospace and other transportation equipment manufacturing
MEM	Electrical machinery and equipment manufacturing
MCC	Computer, communications and other electronic equipment manufacturing
MMI	Instrumentation Manufacturing
OMA	Other manufacturing
UWR	Comprehensive utilization of waste resources
RSM	Repair of metal products, machinery and equipment
PSE	Electricity and heat production and supply industry
PSG	Gas production and supply industry
PSW	Water production and supply industry
